# A single-centre investigator-blinded randomised parallel-group study protocol to investigate the influence of an acclimatisation appointment on children’s behaviour during N_2_O/O_2_ sedation as measured by psychological, behavioural and real-time physiological parameters

**DOI:** 10.1038/s41405-020-0031-y

**Published:** 2020-03-17

**Authors:** Mawlood Kowash, Manal Al-Halabi, Iyad Hussein, Mohammad M. Abdo, Anas Salami, Amar Hassan, Yrsa Sverrisdottir, Jinous F. Tahmassebi

**Affiliations:** 1 Hamdan Bin Mohammed College of Dental Medicine, Mohammed Bin Rashid University of Medicine & Health Sciences, Dubai, United Arab Emirates; 2 College of Medicine, Mohammed Bin Rashid University of Medicine and Health Sciences, Dubai, United Arab Emirates; 3 Leeds School of Dentistry, Leeds University, Leeds, UK

**Keywords:** Dental anxiety and phobia, Dental pharmacology

## Abstract

**Aims and objectives:**

To describe a study protocol of a randomised control trial (RCT) assessing the effectiveness, in reducing dental anxiety, of an acclimatising nitrous oxide sedation (N_2_O) session prior to actual dental treatment with N_2_O.

**Materials and methods:**

A single-centre investigator-blinded parallel-group RCT conducted in a postgraduate dental hospital in Dubai, United Arab Emirates (UAE). Anxious children requiring N_2_O (aged 5–15 years) will be randomly assigned to; a study group: children who will have a preparatory N_2_O trial experience or; a control group: children who will only have N_2_O explained to them. Treatment with N_2_O for both groups will start at the second visit. The following outcomes will be recorded: completion of dental treatment, anxiety scores at baseline and after treatment (using the Modified Child Dental Anxiety Scale faces), behaviour of the child (using Frankl Rating Behaviour Scale) and the acquisition of real-time physiological anxiety-related parameters (using E4^®^ electronic wrist devices).

**Results:**

The data will be analysed statistically.

**Discussion:**

There is a paucity of research regarding dental N_2_O acclimatising appointments. This RCT will supplement existing literature.

**Conclusions:**

This RCT will report whether prior acclimatising of a child to N_2_O sedation is effective, or not, in improving dental treatment behaviour.

## Strengths and limitations

- No previous study has investigated the effect of an N_2_O/O_2_ sedation acclimatisation visit on children’s anxiety and stress levels as measured by psychological, behavioural and real-time physiological parameters.
- This study will provide evidence to support the experts’ opinion regarding the implementation of an acclimatisation visit.
- The study will establish an effective method in the child’s pharmacological behaviour management.
- However, it may expose the child to an additional N_2_O intervention and increase patient contact time which may affect patient compliance.

## Introduction

Nitrous oxide inhalation sedation (nitrous oxide/oxygen—N_2_O/O_2_ or N_2_O for short) is a mild method of anxiolytic sedation. It is a combination of nitrous oxide and oxygen breathed through a snugly fitting nosepiece, using a special delivery device with robust safety features. This aids the anxious child to feel at ease and allow for dental treatment.^1,2^ It is a known fact that fear and anxiety from dentistry are the most commonly known barriers to receiving actual dental treatment,^3^ with millions of patients reporting that they, if offered some type of anxiolysis/sedation, would be more amenable to accepting dental procedures.^4^

The utilisation of N_2_O or general anaesthesia (GA) is advised by contemporary guidelines to enable the performance of dental treatment in children. This is the case when non-pharmacological behaviour shaping methods fail to lessen children’s anxiety and fear,^2^ and where invasive dental treatment (such as extractions and restorations) are needed particularly in countries that have a very high rate of dental decay such as the United Arab Emirates.^5,6^

The application of N_2_O for dental procedures is essentially reliant on the child’s cooperation, allowance of and maintenance of the N_2_O nasal piece to remain securely in position. If N_2_O is gradually titrated to the appropriate levels for the said child, it provides a mild plane of sedation and anxiolysis to allow for dental treatment.^1,2^

It is suggested that the use of an acclimatisation (familiarisation) visit, defined to be a session in which N_2_O sedation is solely provided, with no or minimal dental intervention, would enhance the acceptability and the efficacy of N_2_O success.^2^ This principle may be contentious. It may increase treatment success and compliance, based on the concepts of graded exposure and cognitive behaviour therapy,^7^ which are behind many well-utilised behaviour shaping modalities. However, it may expose the child patient to further pharmacological intercession and increase patient–dentist contact time, subsequently impacting adversely on patient compliance.

The use of an acclimatising N_2_O session had historically been suggested.^8^ While the sedation guidelines of the Royal Colleges of Surgeons and the Royal College of Anaesthetists in the United Kingdom in 2015 proposed two visits for N_2_O—an assessment session without the use of N_2_O—and a treatment session, they recommended N_2_O to be administered at the first visit in cases of dental emergencies only.^9^ Nevertheless, and most recently, other authorities reverted back to the classical viewpoint. For example, the Scottish Dental Clinical Effectiveness Programme (SDCEP) as recent as 2017 recommended the use of an introductory visit stating “*a brief trial of nitrous oxide/oxygen at the assessment appointment may be helpful for the psychological preparation of some children*”.^2^ Therefore, the aforementioned guidelines have advocated the use of an N_2_O accustoming appointment. Nevertheless, and to our awareness, there are no clinical studies that have investigated the effectiveness of such an acclimatisation visit of N_2_O sedation on the acceptance of the child patients of dental procedures. In addition, no robust, methodically designed and objective randomised control trials exist, to our knowledge, to assess if this preparatory appointment would lead to an enhancement in the patients’ performance and behaviour during the dental procedure.

## The aims of the study

This study is a randomised controlled two-arm trial with a single-centre, single-blinded, parallel-group design. The aims of this clinical research project are to assess the outcome and effect of conducting a short acclimatising preparatory appointment, of 15-min duration using N_2_O sedation, on the objectively assessed behaviour of child dental patients and their perceived and measured levels of anxiety during dental treatment. In addition, this study aims to relate and compare this effect in the study group, with that of a group of children who undergo standard dental treatment without the preparatory accustoming appointment of N_2_O sedation.

### The null hypothesis

The employment of an acclimatising appointment of N_2_O sedation, preceding the instigation of dental treatment will not bring about an enhancement in the child’s behaviour and will not lead to a decrease in anxiety levels throughout dental treatment.

## Materials and methods

The outline of the suggested study is that of a single-centre, single-blinded (to dental surgeon and physiologist investigators), parallel-group randomised controlled two-arm clinical trial, adhering to the guidelines of the CONSORT Group (Schulz et al., 2010)^10^ and to reporting checklist for protocol of a clinical trial based on the SPIRIT guidelines. The overall trial start date (from idea conception) is 01/02/2018, and the proposed end date will be 15/10/2020.

### Primary outcome

The changes in the child’s behaviour and anxiety levels. The primary outcome measures will be assessed by

- Physiologically measurable parameters by using a medical grade wearable device (E4^®^ wrist bands—Empatica Inc., 1 Broadway, Cambridge, MA 02142, United States—ISO 13485 Cert. No. 9124.EPTC*)*. These parameters are
  Electrodermal activity (EDA) also known as Galvanic skin response (GSR),
  Blood volume pulse (BVP),
  Heart rate (HR),
  Surface skin temperature.
- Dental anxiety scores at baseline before the (acclimatisation session) and after treatment (second session) using the Modified Child Dental Anxiety Scale-face version (MCDASf).^11^
- Children’s behaviour score will be recorded using the Frankl Behaviour Rating Scale (FBRS).^12^

### Secondary outcomes

The following clinical outcomes will be obtained and considered as indications of a beneficial effect of the acclimatisation N_2_O session:

- The successful completion of the required dental procedure as measured by reviewing the clinical notes documents obtained from the electronic dental records system.
- The children’s and parents’ acceptance of the treatment with or without acclimatisation session as measured by guardian/child quantitative questionnaire (see below Table 1a, b).

**Table 1:** Parent’s (a) and children’s (b) dental treatment acceptance questionnaire.

(a) Parent’s dental treatment acceptance questionnaire					
Statements	Response				
	Strongly agree	Agree	No opinion	Disagree	Disagree Strongly
The dentist explained very well why my child needed dental treatment.					
I have no concerns about how the laughing gas sedation works.					
I think the laughing gas sedation is doing a good job at helping my child to cope with the treatment					
My child coped well with having the laughing gas sedation.					
The dental team were kind and helpful during my child’s treatment.					

(b) Children’s dental treatment acceptance Questionnaire			
Questions	Response		
	Positive	Neutral	Negative
What do you think about your experience with laughing gas?			
Are you glad to have your tooth fixed/extracted?			
How did we look after you when you had your treatment?			
How friendly were we when you came to see us?			
How well did the dentist explain everything about treating your tooth?			
Was it ok having your tooth fixed/extracted?

### Inclusion and exclusion criteria

- Inclusion criteria
  - Children (aged 5–15 years old) presenting with their parents, who are referred to the paediatric dentistry department in Dubai Dental Hospital (DDH) [the only secondary specialist dental hospital in Dubai, United Arab Emirates (UAE)], for dental treatment under N_2_O within a 6 months’ period will be invited to participate in the study.
  - Healthy children indicated for dental treatment under N_2_O with American Society of Anesthesiologists (ASA) classification of I or II.^13^
  - No learning disabilities impeding N_2_O sedation acceptance.
  - UAE and non-UAE nationals’ parents and children will be eligible to participate in the study.
  - The children should have no previous experience of N_2_O inhalation sedation.
- Exclusion criteria
  - Medically compromised children and/or individuals with special healthcare needs.
  - Patients diagnosed with a psychiatric disorder.
  - Children who lack communication due to languages barriers.
  - Children whose parents refuse to participate in the study.
  - Patients with contraindications for the use of N_2_O, such as patients who have upper respiratory tract infections (common cold), mouth breathers due to nasal adenoids.
  - Patients with obvious skin conditions such as eczema or psoriasis that may impede the use of the E4^®^ wrist bands and impair their conductivity.

### Study flow and layout

**Fig. 1:**
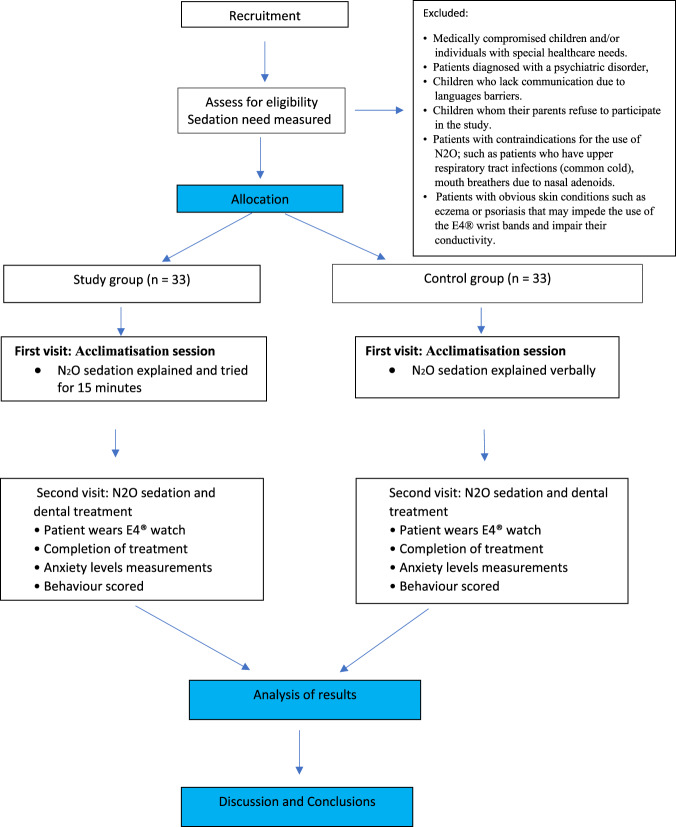
Flowchart of the study design.

A layout of the clinical study is presented in the flowchart seen in Fig. 1. After the initial examination by the clinical investigator, eligibility verification, participant’s assent and parents’/guardian’s signing the informed consent, the study participants will be randomly assigned to either the control or study group (33 patients per group). The study group will include families (children and parents) who are in need of N_2_O sedation at DDH (Dubai, UAE), who would attend a visit for prevention where N_2_O sedation will be introduced and experienced while the control group will be the families who would attend for a prevention visit and discussion of the sedation procedure only without any actual introduction to N_2_O.

A 1:1 allocation-based block randomisation method will be employed. The method of allocation concealment will be attained by using matching, sealed, serially numbered, non-transparent envelopes that will contain group allocation. The envelopes will be opened in sequence by a non-investigator (dental assistant), only after the envelope has been conclusively dispensed to each partaker and will continue to be anonymous to the clinical team.

**Fig. 2:**
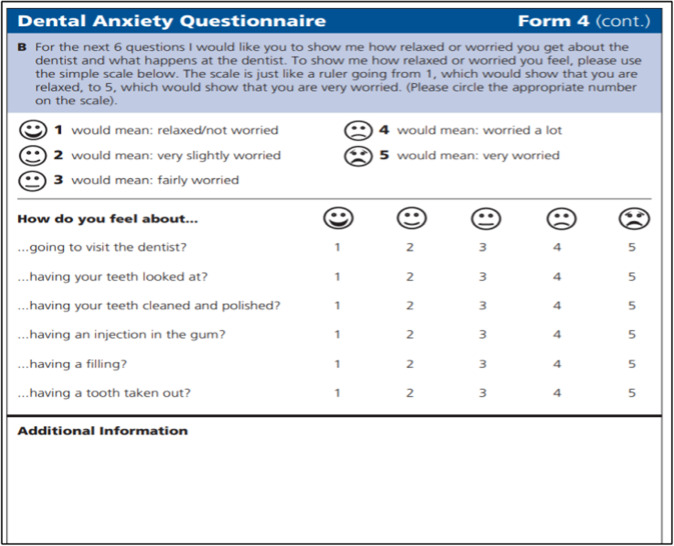
Modified Child Dental Anxiety Scale Faces Version (Source: SDCEP Oral Health Assessment and Review 2017).^11^

**Fig. 3:**
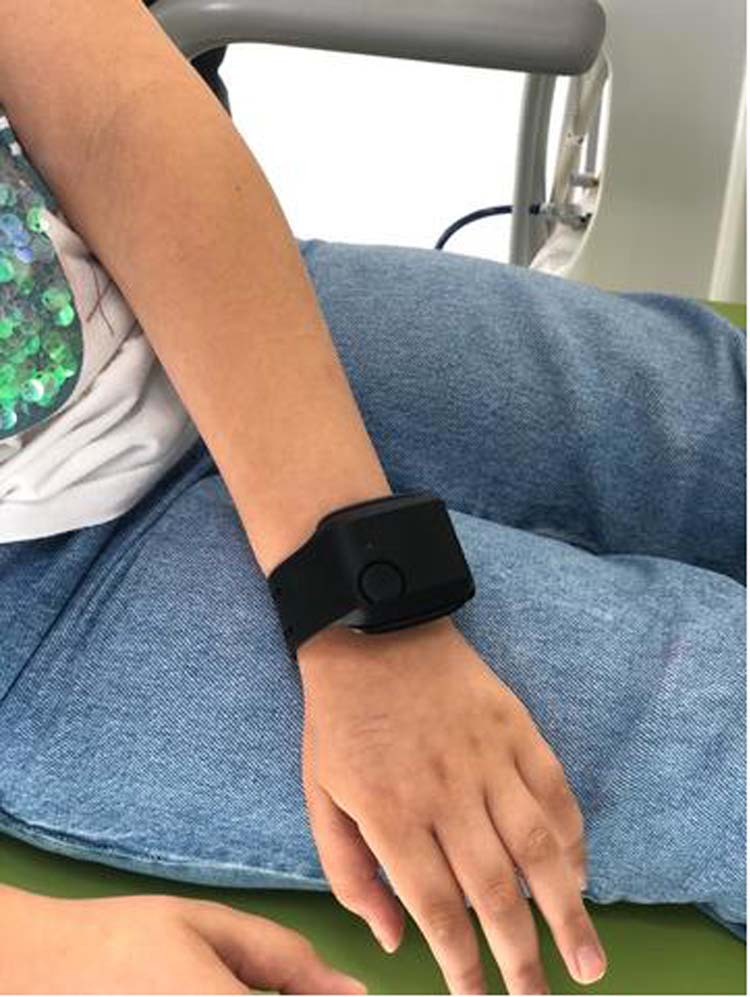
A child wearing the E4 wrist watch.

At the initial screening visit, the anxiety level will be recorded using MCDASf (Fig. 2) to determine the need for N_2_O sedation and subsequently invite the child and parents to participate in the study. Once enroled, an acclimatising visit will be carried out by a single paediatric dentist who will not be involved in the subsequent treatment of the patients. To be constant, an information script will be organised and rehearsed so that the same preoperative instructions about N_2_O sedation will be delivered to all the children and their parents. The patient’s physiological signs will be recorded using the E4^®^ wrist band (Fig. 3). For the study group only, 15 min of N_2_O sedation will be administered without any dental treatment procedures at the acclimatisation visit.

Throughout the second visit, the dental treatment for both study and control groups will include administering local anaesthesia and restoration of carious teeth, pulp treatment if needed and/or tooth/teeth extractions. The dental treatment will be carried out by the same paediatric dentist. However, a second paediatric dentist blinded to which group the child belongs to will record the FBRS of children while receiving the treatment under N_2_O sedation.

The MCDASf (see Fig. 2) using the SDCEP endorsed and condensed version of the MCDASf questionnaire,^11^ not including the last two questions on dental sedation and GA, will be used for all anxious children. The MCDASf is a validated tool that had been used to assess dental anxiety.^14–16^

The language of the MCDASf questionnaire will be English or Arabic. The English version was translated into Arabic using the forward and backward translation method. The translation was checked by an independent bilingual expert who resolved concerns and discrepancies. To make sure that the translation was effective, a back-translation to the English language was done by an independent translator who back-translated the questionnaire and discrepancies in the translation version were resolved. The total score range of MCDASf is 6–30. Children with a score ≥19 will be considered to have severe dental anxiety (dental phobia), while those with a score of <19 will be considered to have none to moderate anxiety (Table 2).^7^ The treatment complexity rank score (Table 3) records the medical status classification, based on the patient’s (ASA) classification.^13^

**Table 2:** Modified Child Dental Anxiety Scores (MCDAS).

Score	Interpretation
5–10	Minimal anxiety
11–18	Moderate anxiety
≥19	Severe anxiety

**Table 3:** Treatment complexity rank score.

Rank	Description	Score
Routine	Polishing, fluoride application, fissure sealants, one-surface Restorations	1
Intermediate	2-surface restorations, extraction of 1 primary tooth, one-quadrant restorative dentistry	2
Complex	Crown preparation, pulp treatment, extraction of multiple primary teeth, multiple-quadrant restorative dentistry, extraction of 1 permanent tooth	3
High complexity	Multiple extractions of permanent teeth, surgical extractions, biopsy. Any treatment considered more complex than above or are multiples of the above	4

**Fig. 4:**
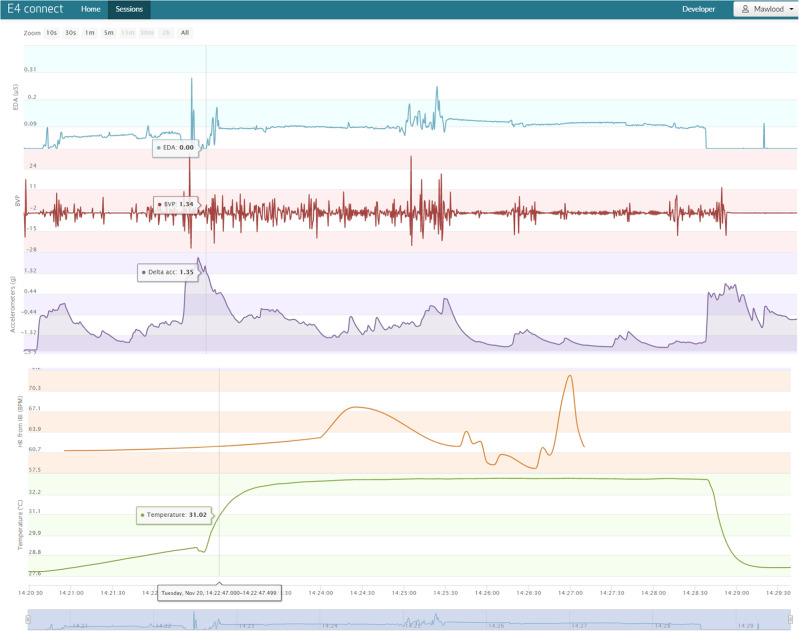
The screenshot from the real-time physiological parameter interface obtained from the E4^®^ watch analytical software.

In addition to the MCDASf, physiological anxiety-related changes will be recorded using E4^®^ wrist bands (see Fig. 3). The wrist band will be placed 5 min before the patient comes into the surgery for treatment and will be taken off 5 min after the completion of treatment. This will enable recording physiological parameters continuously before, during and after the dental treatment. E4^**®**^ wrist bands will provide real-time parameters (Fig. 4) such as EDA also known as GSR, BVP, acceleration, HR, and temperature. E4^**®**^ wrist bands are small, simulate wearing a wristwatch, and we do not anticipate any increase in the children’s anxiety as a result of wearing these wrist bands. The E4^**®**^ wrist band was validated^17^ and approved by the United States Food and Drug Administration as a medical device. It is used in a wide range of research settings with configurations for palmar skin conductance measurement or using gelled electrodes secured under the band. Additional evaluation of behaviour (Table 4) will be documented using the numerical FBRS scores (FBRS: 1–4, where 1 is very uncooperative).^12^ Parents and children will be asked to fill in the survey questionnaire after completion of dental treatment visit to assess their perception and approval (or lack of) of dental treatment with or without an accustoming session (Table 1a, b).

**Table 4:** Frankl behaviour rating scale.^12^

	Frankl behavioural rating scale
Rank	Description
1 − −	Definitely negative: Refusal of treatment, forceful crying, fearfulness or any other overt evidence of extreme negativism.
2 −	Negative: Reluctance to accept treatment, uncooperative, some evidence of negative attitude but not pronounced (sullen, withdrawn).
3 +	Positive: Acceptance of treatment; cautious behaviour at times; willingness to comply with the dentist, at times with reservation, but patient follows the dentist’s directions cooperatively.
4 + +	Definitely positive: Good rapport with the dentist, interest in the dental procedures, laughter and enjoyment.

### Calculation of the study’s sample size

The study is sized to have an 80% power to detect a statistically significant difference in children’s behaviour and anxiety level between the two groups. As there are no previous similar studies, the primary outcome measure is a change in anxiety (a reduction mainly). To our knowledge, no studies had investigated the effect of an acclimatising session of N_2_O on the child’s behaviour to base our sample size calculation on. Our biostatistician used Houpt et al. study in 1996 (effects of nitrous oxide on diazepam sedation of young children) to predict the sample size.^18^ The predicted sample size is 33 participants in each group and to compensate for dropouts a total of 70 participants will be recruited for both groups.

### Role of the participants and the general public in the study

*What was the research question, the outcome and outcome measures of the study and how were they developed?* A pilot questionnaire to explore the usefulness of an N_2_O acclimatising appointment prior to actual treatment, from the participants’ point of view was carried out in a group of 15 child participants (range: 5–15 years). The outcome of this survey was indecisive with 8 out of 15 children expressing a predilection for commencing treatment without an acclimatising session to N_2_O. This resulted in the formulation of the primary outcome measure of our study on investigating the differences in the participants reported dental anxiety scale in children belonging to the study and control groups.

*What were the preferences and experiences of the participants and their influence in designing the study?* We asked the aforementioned children regarding their feeling in carrying out the completion of the MCDASf (anxiety survey). All the children in this survey mentioned that completing MCDASf was acceptable to them. In addition, we showed and placed the E4 watch on their wrists and asked if they would be happy to wear the watch throughout the treatment. Out of 15 children, 13 were happy with wearing the E4 watch; 2 children found it uncomfortable.

*Involvement of participants in the recruitment to and conduct of the study*. The potential participants will already be patients indicated for N_2_O sedation and are on the DDH sedation list.

### Ethical aspects and approval

This study will be conducted in full conformance with principles of the “Declaration of Helsinki”, Good Clinical Practice, and within the laws and regulations of the UAE/Dubai Healthcare City. Participants (legal guardians and children) who meet the aforementioned inclusion criteria will be enroled in this study. An informed consent (verbal and written) will be provided to the parents/caregivers of the child before the N_2_O sedation and enrolment in the study. Partakers and their parents/guardians will be appropriately informed about the set objectives of the study; each participant will be ensured anonymity. Following data analysis, consolidated results will be published. A signed, informed consent form will be required for participation from the parents/caregivers. The Internal Review Board (IRB) committee of Mohammed Bin Rashid University of Medicine and Health Sciences in (MBRU) in Dubai, UAE have approved the study (Reference: MBRU-IRB-2018-014).

### Dissemination of the study’s results

The detailed results and conclusive outcome(s) of the study will be disseminated through peer-reviewed publication/s, conference presentations and the MBRU web site. Additional events will be conducted by inviting stakeholders and groups of interest in inhalation sedation (such as dental teams including general dental practitioners, dental nurses, paediatric dental specialists and consultants to discuss our findings as it will be relevant to all those who manage children who have dental anxiety generally and those using N_2_O specifically).

### The study’s statistical analysis

We will conduct deep statistical analysis after consideration of the data’s distribution normality, with the appropriate parametric or non-parametric statistical tests using the statistical software SPSS™ 24.0.0 (SPSS^©^ Inc., USA). The utilisation of standard descriptive statistics and quantitative data will be reported; if normally distributed, means and standard deviations shall be used; and if skewed, medians and interquartile ranges will be reported. The score of dental anxiety will be calculated for each participant and test for normality by using the Shapiro–Wilk test. Categorical data will be tested for independency by using Chi-square or Fisher's exact test when appropriate. The difference in the means between the two groups will be tested using either *t*-test or Mann–Whitney test depending on the normality of the data. The comparison of the means of more than two variables will be tested by using ANOVA or Kruskal–Wallis depending on the normality of the measurements. A *p*-value of <0.05 will be considered significant in all tests.

## Discussion

The purpose of this study/protocol is to provide an evidence supported answer regarding the clinical value of the use of an additional acclimatising visit for N_2_O prior to the actual planned dental treatment. Nitrous oxide inhalation sedation has been employed in dentistry for over 170 years. Recently, there has been interest for stronger evidence to back its use both generally and in dentistry.^19^ Nevertheless, N_2_O use has been strongly advocated by major international paediatric dentistry organisations. For example, the American Academy of Pediatric Dentistry (AAPD) recognises nitrous oxide/oxygen inhalation as a safe and effective anxiolytic technique proven to reduce dental anxiety that provides mild analgesia and enhances effective communication between a child patient and dental health care provider (AAPD 2015).^1^ The SDCEP (2017) recommends the use of inhalation sedation with nitrous oxide/oxygen as the preferred method for conscious sedation.^2^ These endorsements echo the guidelines of other entities such as the UK dental faculties of the Royal Colleges of Surgeons and the Royal College of Anaesthetists. The latter, in 2015, recommended two visits for inhalation sedation—one preparatory (for assessment purposes for suitability for sedation only) and the other one for the actual treatment.^9^

The employment of an acclimatisation session prior to dental treatment under N_2_O has been suggested by some authors of prominent textbooks in paediatric dentistry since 1980,^8^ under the well-known paediatric dentistry behaviour management principle of Tell–Show–Do.^20,21^ However, the specific advantages of the use of this technique have not been investigated properly.

An additional resource that recommended the use of accustoming visit was the SDCEP in 2017.^2^ They issued the following statement without specific supportive evidence: “*A brief trial of nitrous oxide/oxygen at the assessment appointment may be helpful for the psychological preparation of some children”*. Thus, currently, the guidelines recommendations are based on collective expert opinion and are not evidence based.

The outcomes of this research will provide some evidence on whether or not a separate session for acclimatisation to N_2_O/O_2_ sedation should be offered and whether such extra visit increases the effectiveness of this safe method of sedation to treat anxious children in the clinic. In addition, the requirement of a lengthy time of no oral feeding renders GA a challenge in some young and medically compromised children.^22–24^ Furthermore, dental treatment under N_2_O will decrease treatment costs by about a third, thus avoiding expensive GA hospital admissions.^25^ Although a recent Cochrane report^26^ highlighted that evidence to support the superiority of GA over N_2_O sedation for providing dental care to children is lacking, the safety of the latter is not in question. Our research aims at improving our knowledge in relation to the use of N_2_O, a time-tested method that has cemented its position in the armamentarium available in dealing with anxious dental patients.

### Data statement

Anonymous participants’ data may be shared on request to promote a culture of openness and an increased sharing of research data. Our information sheet and informed consent clearly state that the collected data are intended to be used for research purposes, and possibly published in dental journals and presented at conferences. All the anonymously collected data can be shared upon request from the primary investigator once the study is completed. The data will be stored for 5 years after the final publication to be shared upon request.
